# An International Committee for Graduate Study

**Published:** 1910-04-16

**Authors:** 


### The Hospital

A Journal of

The Medical Sciences and Hospital Administration.

New Series. No. 165, Vol. VII. [No. 1237, Vol. XLVIII.] SATURDAY, APRIL 16, 1910.

### An International Committee for Graduate Study.

The International Committee for Medical Extension Courses, of which we publish the full constitution and membership in this issue, was founded last year at the International Medical Congress held at Budapest. It is a striking testimony to the efficiency of the organisation at the Kaiserin Friedrich Haus, and to the enthusiasm and energy of the General Secretary, that barely six months have elapsed since the preliminaries of the Committee and the actual foundation of the organisation. During these six months Professor Kutner has indeed worked hard, and he thoroughly deserves the congratulations which everyone interested in post-graduate teaching will tender him on the realisation, in a manner which is nothing short of brilliant, of a long-cherished ideal. He has had to contend against that most fearful of discouragements—lukewarmness. He has had to fight the pitiful assertion, "We will see what we can do," and he has had to awaken interest where such interest lay dormant, and to create it in quarters where it was actually non-existent. With such an energetic general secretary the Committee can scarcely suffer shipwreck, and when we wish it cordial prosperity and the best success in its future work, we are fully aware that so long as Professor Kutner presides over the secretariat no effort or pains will be spared and no stone left unturned to make it a practical success in every way.

While we welcome the establishment of the International Committee for Medical Extension Courses, we cannot refrain from pointing out that as at present constituted the English Committee is by no means so truly representative of post-graduate teaching in this country as it should be. A glance at the membership list will show the striking difference between the nominees of the various Continental Governments and those of the English Government. All the former are men who have been actively connected with post-graduate teaching, who are known as lecturers and teachers, and, with few exceptions, have still an active connection with their various schools. None of our nominees, however, are as actively associated with post-graduate schools in this country; none of them, so far as we are aware, have taken any outstanding part in the movement for post-graduate instruction. Dr. Pavy's name is well known in the profession in connection with his researches on diabetes, and he has been a lecturer, both in medicine and in the ancillary sciences, at a London medical school, but he has long ago ceased his connection with that school, and he has never taken any great interest in the post-graduate schools. Dr. Jamieson, who represents Scotland, is an equally well-known teacher, still in active work, and although not permanently connected with any post-graduate school, has lectured to graduate students. He has an intimate acquaintance, too, with the graduate teaching at Vienna. Sir Benjamin Franklin, who is one of the honorary physicians to his Majesty the King, and represents the Indian Medical Service, has had considerable experience of the Paris medical schools, and has himself taken an active interest in post-graduate work. The same can be said of Sir Havelock Charles, who as a lecturer on surgery and morphology was deservedly popular with his post-graduate classes. Both these members, who are representatives of the India Office, are on the retired list, and no longer take any active part in their old schools. Staff-Surgeon Pickthorn, the representative of the Royal Naval post-graduate students, is, on the contrary, in active service and has had experience of post-graduate teaching. As representatives of their special branches in the profession all these gentlemen are excellent; as representatives of the post-graduate students and of the various graduate instruction centres their selection is open to criticism. We trust that the Committee will as soon as possible obviate such criticism by co-opting to its ranks the deans of the various post-graduate schools, and men who, like Sir Jonathan Hutchinson, Dr. Hawthorne, and others, have done so much for the development and advance of graduate teaching in this country.

With this short but, we venture to think, important grumble, we may leave carping, and welcome the Committee as it stands, and its objects and scheme of work as they are set forth. With these latter no fault can be found. The proposed Bureau of Information especially should prove a great boon to intending students, and we would express the hope that the Committee in the near future may see its way clear to publish a short guide to post-graduate study. Such a manual has long been wanted, and from a purely commercial point of view its publication should be a good speculation. The financial question promises to entail some trouble before it is satisfactorily settled, for at present there is no fixed scale of contribution, and Government grants will doubtless be opposed in Parliament by those who object to any expense in connection with matters in which they have no immediate interest. It might be worth while for our graduate schools to take the contribution to be made to the Central Executive Committee upon their shoulders, and so relieve the Government of a charge which, however slight, will always enable the House of Commons to discuss and interfere with the working of the Committee and the general trend of graduate instruction so long as that contribution remains to be provided for out of the Estimates. These, however, are matters which can be fixed when the Committee meets for the first time. For the present let us be grateful that we have a Committee at all. That is a wide step in advance, and one that will inevitably react favourably upon post-graduate education in this country. What is wanted for the success of the new organisation is the loyal help of all those who are interested in such instruction in this country and in the colonies. We have already pointed out that graduate instruction in medicine is not a parochial or purely professional matter; it is a subject of national, of imperial importance, and as such it is to be commended to the notice of everyone who has at heart the interests of the Empire, no less than to those who have at heart merely the welfare of the medical profession.

